# Low-Dose Everolimus Maintenance Therapy for Renal Angiomyolipoma Associated With Tuberous Sclerosis Complex

**DOI:** 10.3389/fmed.2021.744050

**Published:** 2021-11-24

**Authors:** Cong Luo, Wen-Rui Ye, Xiong-Bin Zu, Min-Feng Chen, Lin Qi, Yang-Le Li, Yi Cai

**Affiliations:** ^1^Department of Urology, National Clinical Research Center for Geriatric Disorders, Xiangya Hospital, Central South University, Changsha City, China; ^2^Department of Neurosurgery, Xiangya Hospital, Central South University, Changsha City, China

**Keywords:** tuberous sclerosis complex, renal angiomyolipoma, everolimus, low-dose maintenance therapy, safety

## Abstract

**Objective:** To assess the safety and efficacy of low-dose everolimus maintenance therapy for tuberous sclerosis complex-related renal angiomyolipoma (TSC-RAML) patients that had previously undergone standard-dose treatment for a minimum of 6 months.

**Materials and Methods:** In total, 24 patients with a definitive TSC diagnosis were enrolled from April 2018 – April 2019 at Xiangya Hospital, Central South University. All patients underwent low-dose everolimus maintenance therapy following standard-dose everolimus induction therapy for a minimum of 6 months. Patients additionally underwent TSC1/TSC2 genetic testing, And they were followed-up at 3, 6, 12, 18, and 24 months. The Response Evaluation Criteria in Solid Tumors (RECIST, version 1.1) criteria were used to monitor patient RAML responses, while adverse events (AEs) were assessed as per the National Cancer Institute Common Terminology Criteria for Adverse Events (CTCAE, version 4.0). *P* < 0.05 was the significance level for all analyses, which were performed using SPSS 19.0.

**Results:** TSC1/TSC2 gene mutations were present in all 24 patients, all of whom achieved a significant reduction in TSC-RAML volume within the initial 6-month induction therapy period, and exhibited volume stabilization during the low-dose maintenance therapy treatment period without any instances of TSC-RAML regrowth. Adverse events (AEs) were significantly less severe and less frequent over the course of maintenance therapy relative to standard therapy.

**Conclusions:** Low-dose everolimus maintenance therapy represents an effective approach to achieving TSC-RAML control following a minimum of 6 months of full-dose induction therapy, and may be associated with decreases in everolimus-related AE frequency and severity.

## Introduction

Tuberous sclerosis complex (TSC) is an autosomaldominant syndrome that impacts between 1 in 6,000 and 1 in 10,000 individuals, resulting in characteristic neurodevelopmental features and the development of multiple tumors in organs including the skin, heart, lungs, brain, and kidneys (1). Upwards of 80% of TSC patients are affected by renal angiomyolipoma (RAML) (2), which is characterized by multiple bilateral lesions in the smooth muscles, adipose tissue, and vasculature (3). As these tumors typically grow over time, TSC-RAML can result in arterial hypertension and imposes a risk of life-threatening hemorrhage, which is the leading cause of TSC-associated mortality among adults with this condition (4).

Most TSC patients present with mutations in the *TSC1* or *TSC2* genes, which encode proteins that form the TSC1-TSC2 complex that serves to antagonize the signaling pathway downstream of mammalian target of rapamycin (mTOR) by promoting the activation of the small GTPase Rheb and thereby inhibiting cellular growth and proliferation. Pathogenic TSC1/TSC2 variants result in constitutive mTOR pathway hyperactivation, thereby contributing to the growth of benign tumors or hamartomas in multiple systems (5).

Everolimus is an mTOR inhibitor that has shown promise for the treatment of complications associated with TSC including RAML, seizures, facial angiofibromas, and subependymal giant cell astrocytomas (SEGAs) (6–9). Indeed, everolimus treatment can result in an initial rapid decrease in TSC-RAML volume, followed by a secondary phase during which these tumors slowly shrink or stabilize (7). The International Tuberous Sclerosis Complex Consensus Conference held in 2012 recommended the first-line use of mTOR inhibitors for the treatment of RAML ≥ 3 cm in diameter, even when not associated with any clinical symptoms (10).

Prior work suggests that TSC-RAML regrowth may occur following the cessation of mTOR inhibitor therapy, and the ideal duration for this therapeutic strategy remains to be defined optimal duration of mTOR inhibitor treatment has yet to be determined (11, 12). With respect to safety, the short-term adverse effects associated with everolimus are typically acceptable, although in a few instances more severe events have been reported (13). Long-term treatment-related safety outcomes, however, remain to be established. Wheless and Klim (14) proposed a dose reduction algorithm designed to minimize the negative impact of mTOR inhibitor treatment for patients with SEGAs that are shrinking or stable in size. We were thus interested in whether the everolimus dose could similarly be reduced to control the frequency and severity of adverse events (AEs) in patients with controlled TSC-RAML. This study was therefore designed to examine the safety and efficacy of low-dose everolimus maintenance therapy in TSC-RAML patients that had previously undergone treatment with a standard everolimus dose for a minimum of 6 months.

## Methods

### Study Group

This was a single-center, open-label, single-arm, prospective interventional study performed between April 2018 and April 2019 at Xiangya Hospital, Central South University. The Human Ethics Committee of Xiangya Hospital, Central South University approved this study prior to patient enrollment, and all protocols were performed in accordance with the Declaration of Helsinki (15). Patients provided written informed consent prior to voluntary study participation. Patients eligible for inclusion were: 1) individuals with a definitive TSC diagnosis as defined by meeting 2 major criteria or 1 major criterion and ≥ 2 minor criteria recommended by the 2012 International Tuberous Sclerosis Complex Consensus Conference (10); 2) individuals ≥ 18 years old; 3) individuals with a minimum of one RAML ≥ 30 mm in diameter.

**Figure 1:**
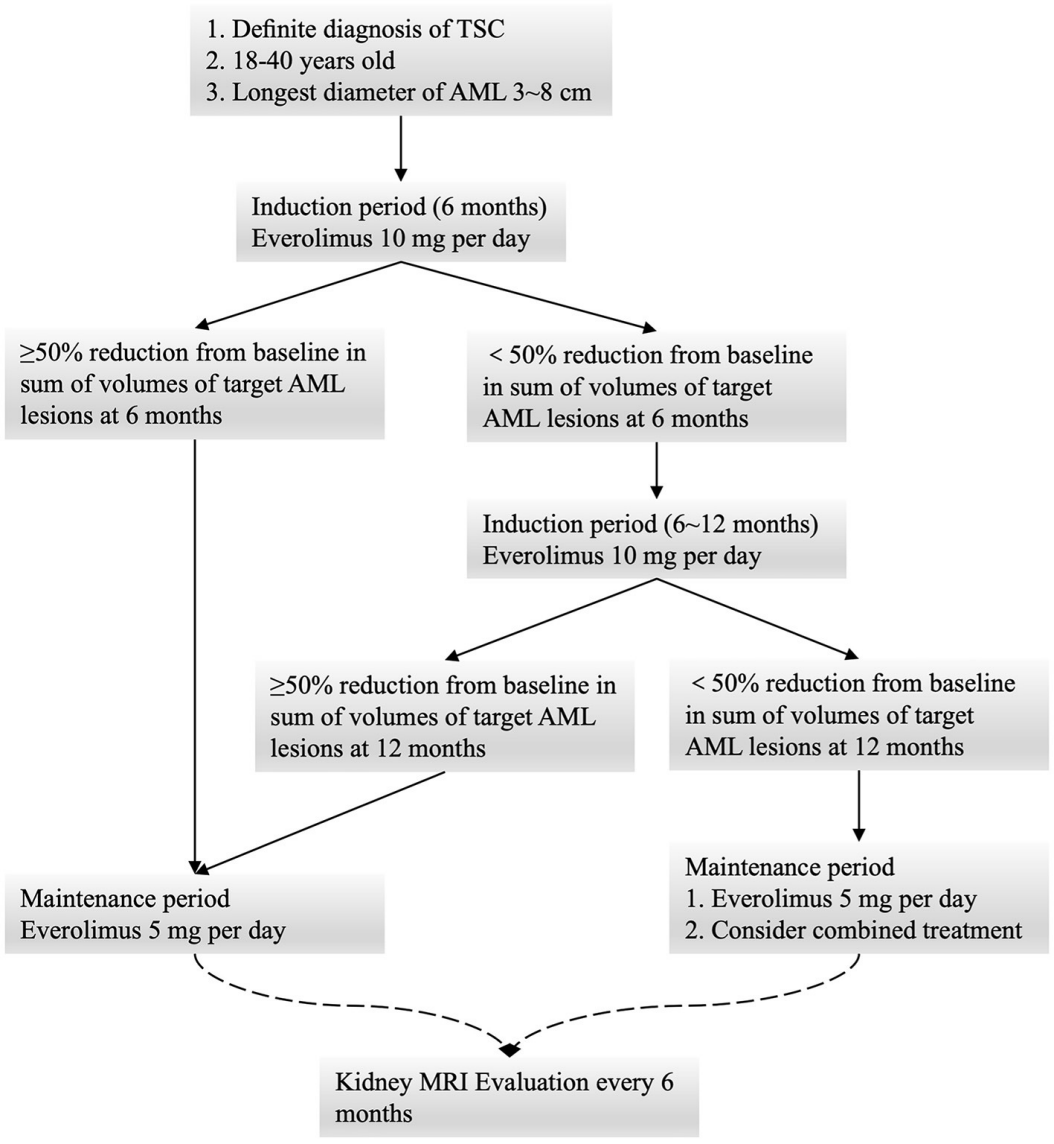
Low-dose maintenance treatment schematic diagram.

All patients underwent oral everolimus induction therapy (10 mg/day) for 6 months, after which they underwent radiographic follow-up and a safety evaluation. All patients that achieved a ≥ 50% decrease in the total volume of the target AML (relative to baseline) were assigned to the low-dose oral everolimus maintenance group (5 mg/day), while patients not meeting these criteria underwent induction therapy for an additional 6 months. Follow-up was then repeated at 12 months, at which time patients were assigned to undergo low-dose maintenance therapy regardless of the observed reduction in AML size. Combination treatment options were considered for individuals exhibiting a poor response to everolimus. Patient follow-up was performed at 3, 6, 12, 18, and 24 months (Figure 1).

### Patient Evaluation and Follow-Up

Abdominal magnetic resonance imaging (MRI) was used to visualize RAML tumors at baseline, with up to four RAMLs with a maximum diameter ≥ 3.0 cm being identified as target lesions in each patient. The sum of the diameters of these target lesions was calculated. Over the course of follow-up, patients underwent routine urine, blood, physical, and radiographic analyses. The Response Evaluation Criteria in Solid Tumors (RECIST, version 1.1) criteria were used to monitor patient RAML responses, while adverse events (AEs) were assessed as per the National Cancer Institute Common Terminology Criteria for Adverse Events (CTCAE, version 4.0).

### Genetic Analysis

TSC1/TSC2 mutational status was assessed via a next-generation sequencing (NGS) approach at the NHC Key Laboratory of Cancer Proteomics (Hunan Province, China). Pathogenic mutations were confirmed through reference to the LOVD databases (www.lovd.nl/TSC1; www.lovd.nl/TSC2). The potential impact of newly identified mutations resulting in amino acid substitutions on protein function was assessed with the online SIFT and PolyPhen2 tools.

### Statistical Analysis

Continuous variables are given as mean ± standard deviation (M ± SD), while categorical variables are given in the form of frequencies (n) and percentages (%). Categorical variables were compared using the chi-square test. SPSS 19.0 (SPSS, IL, USA) was used for all statistical testing, with P < 0.05 as the significance threshold.

## Results

### Patient Characteristics

In total, 24 patients (8 male, 16 female) were enrolled in this study, with a median age of 27 years. Patient demographics are compiled in Table 1. Of these patients, 20 and 4 were found to harbor *TSC2* and *TSC1* mutations, respectively, via NGS (Table 2). Three of these patients had a history of epilepsy, two were treated with antiepileptic monotherapy (oxcarbazepine, lamotrigine), while the remaining one with antiepileptic combination therapy (oxcarbazepine + topiramate). Five of these patients were diagnosed with lymphangioleiomyomatosis (LAM), all suffered from skin lesions, and one presented with SEGAs. One patient had a renal impairment of GFR < 60 mL/min. With respect to the baseline RAML characteristics in these patients, 11 exhibited RAMLs with a maximum diameter ≥ 6 cm, 20 exhibited bilateral RAMLs, and 10 presented with 3-4 target RAML lesions.

**Table 1:** Baseline patient demographic and disease characteristics.

	**Everolimus (*N* = 24)**
Age in years, median (range)	27 (19–33)
<30	18 (75%)
≥30	6 (25%)
Sex	
Male	8 (33.3%)
Female	16 (66.7%)
Gene mutation	
TSC1	4 (16.7%)
TSC2	20 (83.3%)
Epilepsy	3 (12.5%)
Diagnosis of LAMs	5 (20.8%)
Skin lesions (≥1)	24 (100%)
Presence of SEGAs	1 (4.1%)
Renal impairment (GFR <60 mL/min)	1 (4.1%)
Diameter of the largest RAML lesions	
6–8 cm	11 (45.8%)
4–6 cm	11 (45.8%)
3–4 cm	2 (8.3%)
Sum of volumes of target renal angiomyolipoma lesions, cm^3^	
Mean (SD, cm^3^)	155.7 (100.4)
Median (range, cm^3^)	121.6 (29.6~348)
Bilateral angiomyolipoma	20 (88.3%)
Number of target RAML lesions	
1~2	14 (58.3%)
3~4	10 (41.7%)
Previous angiomyolipoma therapy	
Surgery/invasive procedure	9 (37.5%)
Renal embolization	3 (12.5%)
Partial nephrectomy	2 (8.3%)
Nephrectomy	4 (16.7%)
Medication	0 (0)

*LAMs, lymphangioleiomyomatosis; SEGAs, subependymal giant cell astrocytomas; RAML, renal angiomyolipoma*.

**Table 2:** Mutations detected by next-generation sequencing.

**No**	**Sex**	**Age**	**Mutant gene**	**Nucleotide change**	**Protein change**	**Mutation Type**
1	Female	19	TSC2	c.1922G>T	Ser641Ile	Missense mutations
2	Female	22	TSC2	c.2407C>T	Gln803^*^	Nonsense mutations
3	Female	23	TSC2	c.2407C>T	Gln803^*^	Nonsense mutations
4	Female	24	TSC2	c.3838G>T	Gln1280^*^	Nonsense mutations
5	Male	24	TSC2	c.5161del	Met1721Trpfs^*^105	Frameshift mutations
6	Male	27	TSC2	c.1001T>G	Val334Gly	Missense mutations
7	Female	28	TSC1	c.1431_1434del	Glu478Lysfs^*^53	Frameshift mutations
8	Female	29	TSC2	c.2098-2A>G	p?	Intron mutation
9	Male	32	TSC2	c.3707T>C	Met1236Thr	Missense mutations
10	Female	24	TSC1	c.1960C>T	Gln654^*^	Nonsense mutations
11	Male	26	TSC2	c.2785G>T	Glu929^*^	Nonsense mutations
12	Female	26	TSC2	c.1348G>T	Glu450^*^	Nonsense mutations
13	Female	25	TSC1	c.1960C>T	Gln654^*^	Nonsense mutations
14	Male	26	TSC2	c.5024C>T	Pro1675Leu	Missense mutations
15	Male	26	TSC2	c.820T>A	Tyr274Asn	Missense mutations
16	Female	27	TSC1	309G>A	Trp103^*^	Nonsense mutations
17	Female	28	TSC2	c.2251C>T	Arg751^*^	Nonsense mutations
18	Male	29	TSC2	c.2988del	Ser997Valfs^*^19	Frameshift mutations
19	Female	29	TSC2	c.4604A>T	Asp1535Val	Missense mutations
20	Male	30	TSC2	c.3180G>A	Trp1060^*^	Nonsense mutations
21	Female	31	TSC2	c.3412C>T	Arg1138^*^	Nonsense mutations
22	Female	31	TSC2	c.4708A>T	Arg1570Trp	Missense mutations
23	Female	33	TSC2	c.1547_1559delinsGTGCTGCC	Ala516Glyfs^*^71	Frameshift mutations
24	Female	33	TSC2	EX25_36 DEL	–	Large rearrangements

Before enrollment in this study, 3, 2, and 4 of these patients had respectively undergone renal embolization, partial nephrectomy, and nephrectomy. No patients had undergone prior medication therapy. Over the follow-up period, two patients withdrew from the study at 24 months, leaving 22 patients for the assessment of RAML status. One gave up medication for economic reason, and the other withdrew from the study due to the unavailability of everolimus resulted from COVID-19 pandemic.

### Treatment Efficacy

**Figure 2:**
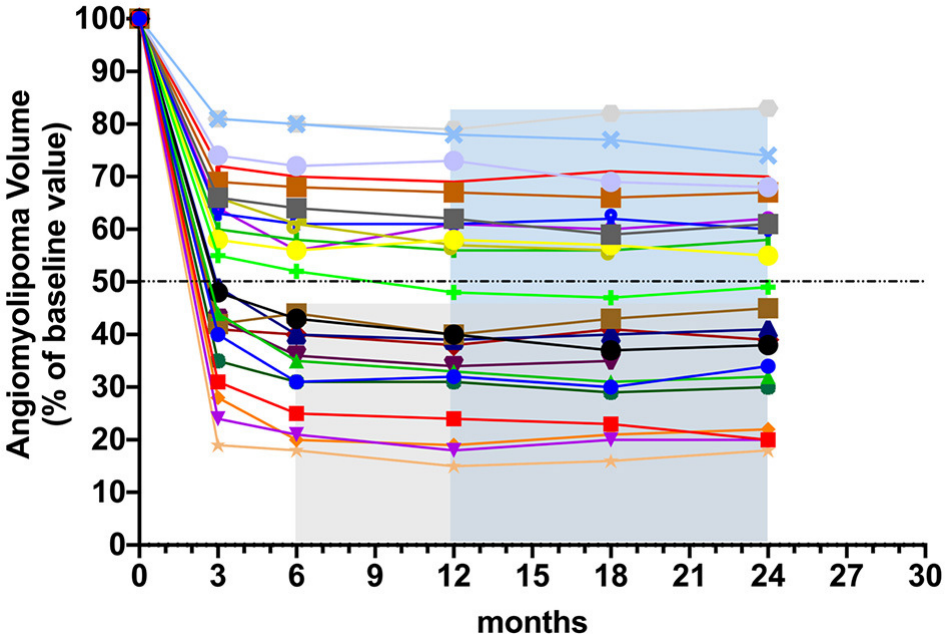
Changes in TSC-RAML volume from baseline during the induction therapy and the maintenance therapy. Each line represents TSC-RAML volume change in one patient.

The patients' response of AML volume to everolimus during treatment is detailed in Table 3. The number of patients who achieved ≥ 50% reduction in RAML volume was 12 (50%) at 6 months and 13 (54%) at 12 months, respectively. The change in RAML volume for each patient over the study period is displayed in Figure 2, with the most significant decrease in tumor volume having been observed within the initial 6 months of standard-dose everolimus therapy. In total, 12 patients achieved ≥ 50% reduction in total target AML volume at 6 months, whereupon they initiated low-dose everolimus maintenance therapy. Just one of the remaining 12 patients achieved ≥ 50% reduction in target AML volume after an additional round of full-dose everolimus treatment. Target RAML volumes were well-controlled in all patients during the maintenance therapy period.

**Table 3:** Response of AML volume to everolimus therapy.

	**3 months**	**6 months**	**12 months**	**18 months**	**24 months**
Patients (*n*)	24	24	24	24	22
No. of response (*n*, %)	12 (50)	12 (50)	13 (54)	13 (54)	12 (55)
^*^% of baseline value (Mean ± SD, %)	48 ± 18	52 ± 19	53 ± 20	53 ± 19	52 ± 19

* *The average percentage change of baseline in the total volume of all target AML lesions*.

**Figure 3:**
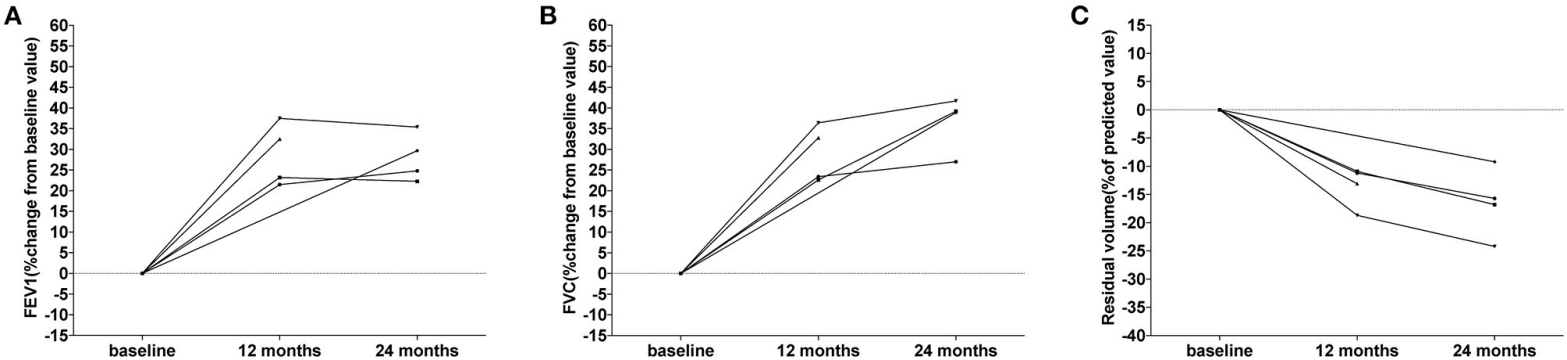
Changes of pulmonary function in the five patients with LAM. **(A)** The changes of FEV1 from baseline during treatment. Each line represents FEV1 change in one patient. **(B)** The changes of FVC from baseline during treatment. Each line represents FVC change in one patient. **(C)** The changes of residual volume from baseline during treatment. Each line represents residual volume change in one patient.

The pulmonary function in 5 female patients with LAM during everolimus therapy are detailed in Table 4. At baseline, four patients showed moderate airflow obstruction (forced expiratory volume in 1 second (FEV1): 50–70% of the predicted value), while one patient showed severe airflow obstruction (FEV1 < 50% of the predicted value). During the medication period, an increase was observed in FEV1, forced vital capacity (FVC), total lung capacity, and diffusion capacity for carbon monoxide (DLCO) among all the five patients, while the residual volume decreased. The changes in FEV1, FVC, and residual volume for each patient are shown in Figure 3. It further displayed the improved pulmonary function in patients with LAM. These results indicated that both full-dose and low-dose everolimus treatment have a protective effect on pulmonary function in patients with TSC.

**Table 4:** Pulmonary functional characteristics of patients with LAM.

**Value**	**Baseline**	**12 months**	**24 months**
	**(*n* = 5)**	**(*n* = 4)**	**(*n* = 4)**
FEV1			
Least-square mean (liters)	1.30 ± 0.15	1.63 ± 0.27	1.69 ± 0.29
Percent of predicted value	55.08 ± 9.26	62.06 ± 10.09	73.02 ± 14.93
FVC			
Least-square mean (liters)	2.52 ± 0.54	3.02 ± 0.62	3.67 ± 0.72
Percent of predicted value	73.29 ± 12.37	85.52 ± 12.13	100.39 ± 11.37
Total lung capacity			
Least-square mean (liters)	4.80 ± 0.52	5.18 ± 0.29	5.91 ± 0.53
Percent of predicted value	92.09 ± 6.64	100.23 ± 4.22	112.92 ± 9.13
Residual volume			
Least-square mean (liters)	2.28 ± 0.07	1.97 ± 0.14	1.90 ± 0.18
Percent of predicted value	120.40 ± 5.35	111.72 ± 6.69	112.30 ± 7.06
DLCO			
Least-square mean (ml/mmHg/min)	12.03 ± 2.52	12.70 ± 2.62	15.34 ± 2.10
Percent of predicted value	49.43 ± 9.53	52.13 ± 8.01	62.08 ± 9.35

*FEV1, forced expiratory volume in 1 s; FVC, forced vital capacity; DLCO, diffusion capacity for carbon monoxide*.

### Adverse Events

All AEs recorded during the induction and maintenance phases of everolimus treatment are compiled in Table 5. While six grade 3–4 AEs occurred during full-dose induction therapy, none occurred during low-dose maintenance therapy. The most common AEs during full-dose induction therapy included oral mucositis (22/24), abdominal pain (10/24), hypertriglyceridemia (10/24), and headache (9/24), while the most common AEs during low-dose maintenance treatment were oral mucositis (10/24) and hypertriglyceridemia (9/24). AEs with significant incidence reductions during low-dose maintenance therapy included oral mucositis (*P* < 0.001), irregular menstruation (*P* = 0.04), upper respiratory infections (*P* = 0.02), and fever (*P* = 0.04). No unexpected AEs or mortality were reported, and no patients declined treatment or withdrew from the study due to AEs.

**Table 5:** Adverse events associated with everolimus in the study group during induction and maintenance therapy.

**Adverse events**	**Induction therapy**		**Maintenance therapy**		***P-*value**
	**All grade**	**Grade 3/4**	**All grade**	**Grade 3/4**	
Mucositis oral	22/24	0	10/24	0	**<0.001**
Irregular menstruation	8/16	4/16	1/16	0	**0.04**
Abdominal pain	10/24	0	5/24	0	0.12
Hypertriglyceridemia	10/24	0	9/24	0	0.77
Headache	9/24	0	6/24	0	0.35
Diarrhea	8/24	0	7/24	0	0.76
Upper respiratory infection	7/24	0	1/24	0	**0.02**
Proteinuria	6/24	1/24	5/24	0	0.73
Malaise	6/24	0	5/24	0	0.73
Rash acneiform	5/24	1/24	2/24	0	0.22
Cholesterol high	5/24	0	3/24	0	0.44
Fever	4/24	0	0	0	**0.04**
Urinary tract infection	4/24	0	2/24	0	0.38
Hematuria	3/24	0	0	0	0.07
Alkaline phosphatase increased	3/24	0	4/24	0	0.68
Constipation	3/24	0	1/24	0	0.30
GGT increased	3/24	0	2/24	0	0.64
Hypophosphatemia	3/24	0	1/24	0	0.30
Seizures	2/24	0	0	0	0.15
Pneumonitis	2/24	0	0	0	0.15
Vomiting	2/24	0	0	0	0.15
Lymphocyte count decreased	2/24	0	1/24	0	0.55
Anemia	2/24	0	2/24	0	1
Neutrophil count decreased	1/24	0	0	0	0.31
Hyperuricemia	1/24	0	0	0	0.31
Creatinine increased	1/24	0	1/24	0	1

*The bold values refers to p < 0.05 with statistic significance*.

## Discussion

This study is the first to our knowledge to have assessed the efficacy and safety of low-dose everolimus maintenance therapy for the treatment of TSC-RAML patients after a minimum 6-month full-dose induction therapy period.

Owing to the potential for rebound after withdrawal, sustained everolimus therapy is necessary to effectively control TSC-RAML. However, continuous everolimus treatment is associated with a number of issues. First, prolonged standard everolimus treatment is expensive and can impose a major economic burden on patients that decreases their compliance. Second, lifelong mTOR inhibitor treatment is often required for TSC patients, particularly for individuals < 40 years of age, emphasizing the need to explore more feasible or cost-effective solutions. Third, sustained standard everolimus treatment can result in potentially severe AEs. Lastly, the most prominent tumor growth reduction generally occurs within the initial 3–6 months of treatment in patients, whereafter tumor volumes tend to stabilize or decrease gradually (16). We therefore designed the present study to assess the ability of low-dose everolimus maintenance to control RAML volumes and to reduce AE incidence, given that such an approach has previously been reported to be successful in the treatment of TSC-related SEGAs (17).

Herein, the everolimus dose utilized for low-dose maintenance therapy was reduced to 5 mg/day from 10 mg/day. All patients achieved a significant reduction in TSC-RAML volume over the first 6 months of induction treatment, and maintained a stable TSC-RAML volume during the low-dose maintenance period without any evidence of target AML lesion growth or progression. This suggests that low-dose everolimus maintenance therapy is an effective therapeutic option for TSC-AML patients. Moreover, pulmonary functions, including FEV1, FVC, total lung capacity, DLCO, and residual volume, were improved in five patients with LAM during both full- and low-dose everlimus therapy, which further confirmed the efficacy of low-dose everlimus therapy.

Everolimus therapy is commonly associated with a range of AEs that can affect treatment efficacy and compliance in some patients. We found that these everolimus therapy-related AEs were significantly less frequent and less severe during the low-dose maintenance therapy compared with the standard treatment period, consistent with a previous study (17). Reducing the incidence of oral mucositis is critical to improving patient compliance. Most importantly, no grade 3-4 AEs were observed in the context of low-dose maintenance therapy, in contrast to the incidence of such complications during full-dose everolimus treatment. These results thus suggest that low-dose everolimus maintenance therapy is a feasible and well-tolerated option for patients with TSC-RAML.

While these results are promising, this study is nonetheless limited by the fact that it is a single-center analysis of a relatively small patient population. Even so, we hope that this study can provide a reference for the everolimus treatment of TSC-RAML patients, particularly those patients that exhibit poor tolerance for full-dose everolimus therapy. We plan to recruit more patients for treatment with this low-dose maintenance therapy regimen, and as such, our available efficacy and safety data will continue to expand in the future. Another noteworthy limitation of this study is that we were unable to assess drug concentrations in patient blood owing to technical limitations. However, we believe that consistent dosing will largely mitigate the potential bias associated with this limitation.

## Conclusions

Low-dose everolimus maintenance therapy is an effective therapeutic approach to controlling TSC-RAML following full-dose induction therapy, and may reduce the frequency and severity of AEs associated with everolimus.

## Data Availability Statement

The original contributions presented in the study are included in the article/Supplementary Material, further inquiries can be directed to the corresponding authors.

## Ethics Statement

The studies involving human participants were reviewed and approved by the Institutional Review Board of Xiangya Hospital, Central South University. The patients/participants provided their written informed consent to participate in this study.

## Author Contributions

CL, W-RY, Y-LL, and YC participated in its design and coordination and drafted the manuscript. CL, W-RY, and Y-LL participated in the design of the study and performed the statistical analysis. CL, W-RY, M-FC, LQ, X-BZ, Y-LL, and YC conceived of the study, participated in its design, and coordination and helped to draft the manuscript. All authors have read and approved the final manuscript.

## Funding

This research was supported by the National Natural Science Foundation of China (Grant No. 81800590) and the Natural Science Foundation of Hunan Province (Grant No. 2020JJ5882).

## Conflict of Interest

The authors declare that the research was conducted in the absence of any commercial or financial relationships that could be construed as a potential conflict of interest.

## Publisher's Note

All claims expressed in this article are solely those of the authors and do not necessarily represent those of their affiliated organizations, or those of the publisher, the editors and the reviewers. Any product that may be evaluated in this article, or claim that may be made by its manufacturer, is not guaranteed or endorsed by the publisher.
