# Supplementary material for: Low-Dose Everolimus Maintenance Therapy for Renal Angiomyolipoma Associated With Tuberous Sclerosis Complex
Source: Front Med (Lausanne). 2021 Nov 24;8:744050. doi: 10.3389/fmed.2021.744050 (PMC8652067; doi:10.3389/fmed.2021.744050)
Supplement: Supplementary file 2 [file Table_2.DOCX]

**Supplementary Table**

| **No.** | **patient** | **Baseline (V)** | **12 m (%)** | **24 m (%)** |
| --- | --- | --- | --- | --- |
| FEV1  Least-square mean (95% CI) — liters | No. 7 | 1.21 | 1.47 | 1.51 |
|  | No. 19 | 1.12 | 1.38 | 1.37 |
|  | No. 22 | 1.26 | 1.67 | - |
|  | No. 23 | 1.44 | 1.98 | 1.95 |
|  | No. 24 | 1.48 | - | 1.92 |
| FEV1  Percent of predicted value (95% CI) | No. 7 | 52.45 | 56.9 | 62.12 |
|  | No. 19 | 53.4 | 58.34 | 59.24 |
|  | No. 22 | 42.19 | 55.89 | - |
|  | No. 23 | 60.56 | 77.12 | 80.06 |
|  | No. 24 | 66.80 | - | 90.65 |
| FVC  Least-square mean (95% CI) — liters | No. 7 | 2.52 | 3.11 | 3.20 |
|  | No. 19 | 2.12 | 2.60 | 2.95 |
|  | No. 22 | 1.89 | 2.51 | - |
|  | No. 23 | 2.83 | 3.86 | 4.01 |
|  | No. 24 | 3.24 | - | 4.50 |
| FVC  Percent of predicted value (95% CI) | No. 7 | 73.11 | 93.58 | 96.25 |
|  | No. 19 | 64.60 | 77.94 | 88.33 |
|  | No. 22 | 58.40 | 72.65 | - |
|  | No. 23 | 81.16 | 97.89 | 101.62 |
|  | No. 24 | 89.20 | - | 115.35 |
| Total lung capacity  Least-square mean (95% CI) — liters | No. 7 | 4.75 | 5.28 | 5.43 |
|  | No. 19 | 4.49 | 5.17 | 5.85 |
|  | No. 22 | 4.18 | 4.79 | - |
|  | No. 23 | 5.02 | 5.49 | 5.70 |
|  | No. 24 | 5.54 | - | 6.66 |
| Total lung capacity  Percent of predicted value (95% CI) | No. 7 | 91.20 | 100.85 | 103.73 |
|  | No. 19 | 88.89 | 100.57 | 113.98 |
|  | No. 22 | 83.68 | 94.63 | - |
|  | No. 23 | 95.47 | 104.88 | 108.88 |
|  | No. 24 | 101.21 | - | 125.10 |
| Residual volume  Least-square mean (95% CI) — liters | No. 7 | 2.23 | 1.98 | 1.88 |
|  | No. 19 | 2.38 | 2.12 | 1.98 |
|  | No. 22 | 2.29 | 1.99 | - |
|  | No. 23 | 2.19 | 1.78 | 1.66 |
|  | No. 24 | 2.29 | - | 2.08 |
| Residual volume  Percent of predicted value (95% CI) | No. 7 | 116.78 | 108.63 | 111.74 |
|  | No. 19 | 126.07 | 118.89 | 114.74 |
|  | No. 22 | 121.89 | 115.38 | - |
|  | No. 23 | 113.12 | 103.98 | 102.94 |
|  | No. 24 | 124.16 | - | 119.76 |
| DLCO  Least-square mean (95% CI) —ml/mmHg/min | No. 7 | 12.12 | 13.42 | 13.80 |
|  | No. 19 | 10.24 | 11.79 | 13.36 |
|  | No. 22 | 8.89 | 9.69 | - |
|  | No. 23 | 13.89 | 15.90 | 16.50 |
|  | No. 24 | 15.02 | - | 17.70 |
| DLCO  Percent of predicted value (95% CI) | No. 7 | 47.24 | 53.79 | 55.33 |
|  | No. 19 | 43.97 | 47.61 | 53.95 |
|  | No. 22 | 37.92 | 44.46 | - |
|  | No. 23 | 56.51 | 62.65 | 65.04 |
|  | No. 24 | 61.52 | - | 73.99 |
